# Sociodemographic disparities in sedentary time among US youth vary by period of the day

**DOI:** 10.1371/journal.pone.0296515

**Published:** 2024-01-05

**Authors:** María Enid Santiago-Rodríguez, Jinsong Chen, Karin A. Pfeiffer, David X. Marquez, Eduardo Esteban Bustamante

**Affiliations:** 1 School of Kinesiology, University of Michigan, Ann Arbor, Michigan, United States of America; 2 School of Public Health, University of Nevada Reno, Reno, Nevada, United States of America; 3 College of Medicine, University of Illinois, Chicago, Illinois, United States of America; 4 Department of Kinesiology, Michigan State University, East Lansing, Michigan, United States of America; 5 Department of Kinesiology & Nutrition, University of Illinois Chicago, Chicago, Illinois, United States of America; University of Balearic Island, SPAIN

## Abstract

**Introduction:**

Studies have reported sex and race/ethnicity disparities in sedentary time (ST), but none have evaluated ST by well-defined periods of the weekday (before school, during school, afterschool, and evening) and weekend day (morning, afternoon, and evening). Comparing sex and race/ethnicity disparities in ST at different periods of a weekday and weekend day can deepen our understanding of disparities and inform intervention efforts. This study tests sex and race/ethnicity disparities in ST by period of day in a representative sample of US youth.

**Methods:**

Youth (N = 2,972) from the 2003–2006 NHANES waves reported demographic variables and wore an accelerometer for 7 consecutive days to assess ST. Linear regressions were conducted to test relationships between sex and race/ethnicity and ST (min/hour) during each period of a weekday and weekend day. ST differences by sex and race/ethnicity were calculated to identify the periods of the day presenting the largest opportunity to reduce disparities.

**Results:**

Females were more sedentary than males during school (p < 0∙0001), afterschool (p < 0∙0001), and weekday evenings (p < 0∙0001) after controlling for covariates. After controlling for covariates, race/ethnicity only was a significant predictor of ST during weekend mornings (p < 0∙0001). During school and afterschool emerged as the periods with the largest opportunities to reduce sex disparities in ST. Weekend mornings were identified as the largest opportunity to reduce race/ethnic disparities in ST.

**Conclusions:**

Sex disparities in ST appear to be driven mostly by the during school period of the day, while race/ethnic disparities in ST seem to be driven by the weekend morning period. Future intervention work should consider these periods when aiming to reduce ST disparities in youth.

## Introduction

Cross-sectional studies regarding self-reported and accelerometer assessed sedentary time (ST) have reported that ST varies by sex [1] and race/ethnicity [2]. For instance, Matthews et al. (2008) used NHANES accelerometer data from the 2003–2004 wave to compare ST by race/ethnicity and age [2]. The authors reported that only Mexican Americans from 16–19 years were less sedentary compared to non-Hispanic Whites. Disparities in device-based ST have also been reported by sex; cross-sectional data from 6,539 children from Australia, Brazil, Canada, China, Colombia, Finland, India, Kenya, Portugal, South Africa, United Kingdom, and United States (US) showed that girls (521 min/day) spent more time being sedentary than boys (504 min/day) [1]. These studies establish sociodemographic disparities in device-based ST, but they do not provide insight into the periods of the day driving youth ST disparities. This is a missed opportunity, given that an understanding of the periods of the day during which disparities occur could provide meaningful information to interventionists. Youth routines are predictable and at different periods of the day youth are with different adults, engaged in different activities, in different locations. Given the context dependent nature of intervention implementation, understanding the periods of the day during which disparities emerge may assist interventionists in selecting settings that will most reduce sociodemographic disparities in ST.

To date, two studies have examined sociodemographic disparities in ST by periods of the day (i.e., before school, during school, and evening) [3, 4]. Kallio et al. evaluated sex differences in ST in a sample of 970 children using accelerometers [3]. The authors found that, over a 2-year period, ST significantly increased during out of school hours and school hours for boys and girls. However, the ST increase was larger for boys. The authors did not define distinct time periods for out of school hours, so it remains unknown whether this change was occurring before school, after school, or during the evenings. The other study recruited 2,225 children and adolescents to evaluate ST via accelerometry at different periods such as weekend, time out-of-school weekdays, and during school [4]. Authors reported that girls were more sedentary than boys during all periods of the day [4]. Although these studies were conducted in Spain [4] and Finland [3], cross-sectional data about ST from different parts of the world support that females are more likely to be more sedentary than males regardless of the country [1].

ST varies by race/ethnicity and sex, but gaps in the literature aside from those related to time of day remain. No studies have utilized a representative sample of US youth, nor a sample large enough to accommodate analyses with numerous important covariates. By addressing these limitations, a more fine-grained understanding about ST patterns related to periods of the day will be available for interventionists to use in planning. Thus, the purpose of this study was to evaluate sex and racial/ethnic disparities in device-based ST among youth in the US by period of the day. This was accomplished by analyzing two waves of data from the National Health and Nutrition Examination Survey (NHANES) 2003–2006. The aim of this study was to compare ST at different periods of an average weekday (before school, during school, afterschool, and evening) and weekend day (morning, afternoon, and evening) by sex (male, female) and race/ethnicity (non-Hispanic White, Mexican American, Other Hispanic, non-Hispanic Black, and Other Race–Including Multi-Racial).

## Methods

### National Health and Nutrition Examination Survey

Data from the National Health and Nutrition Examination Survey (NHANES) were used to conduct a secondary analysis of cross-sectional data to test sex and race/ethnic disparities in ST by periods of the week and weekend day. NHANES measures and protocol have been explained elsewhere [5]. This survey examines a nationally representative sample of ~5,000 participants in the US every year on physical health and lifestyle behaviors in children and adults through interview and physical examination [6]. Demographics, socioeconomic status, and lifestyle behavior data are obtained through the interview. The physical examination gathers information about physical health such as laboratory tests, medical, dental, and physiological information.

### Participants

The eligibility criteria for the present study sample consisted of any youth who participated in the 2003–2004 and 2005–2006 waves of NHANES and were between 6 and 18 years of age. The classifications for race/ethnicity were based on the NHANES dataset. Any participant that reported being a Mexican American, regardless of other race/ethnicity identity, were classified as Mexican American. Those participants who self-identified as Hispanics, but not as Mexican American, were coded as other Hispanics. Participants who identified themselves as non-Hispanics were coded as non-Hispanic white if they selected white as their main race or non-Hispanic Black if they selected black as their main race. However, for those who identified themselves as non-Hispanics and indicated more than one race (multiracial) or any other race besides white and black, then they were coded as other race- including multiracial. The study was exempt of Institutional Review Board’s approval since it did not meet the definition of human subject research because it only involved the analysis of publicly available data. For the current analysis, the dataset was accessed on January 2019. Table 1 in the appendix presents the participants distribution by sex and race/ethnicity.

**Table 1:** Regression of sedentary time by periods of the week day and weekend day on sex.

		Unadjusted Model		Adjusted Model		
Period of the Day	Variables	Beta (95% CI)	P	Beta (95% CI)	P	*R* ^2^
Before School	Female	0.6 (-1.5, 2.7)	0.5545	-	-	-
During School	Female	**3.2 (2.4, 4.0)**	**< 0.0001**	**2.8 (2.2, 3.5)**	**<0.0001**	**0.27**
Afterschool	Female	**2.5 (1.5, 3.5)**	**< 0.0001**	**2.1 (1.3, 2.9)**	**<0.0001**	**0.29**
Weekday Evening	Female	**1.8 (0.8, 2.8)**	**0.001**	**1.4 (0.5, 2.4)**	**0.004**	**0.23**
Weekend Morning	Female	**2.1 (1.1, 3.2)**	**0.0002**	**1.7 (0.7, 2.7)**	**0.002**	**0.15**
Weekend Afternoon	Female	**2.1 (1.2, 3.1)**	**<0.0001**	**1.7 (0.9, 2.4)**	**<0.0001**	**0.01**
Weekend Evening	Female	1.4 (0.3, 2.6)	0.02	-	-	-

**Notes**. In the adjusted model, the following covariates were adjusted: race, age, income, and BMI. An alpha level of <0.004 was used to determine significance.

### Variables

ST was obtained via accelerometry. Participants were instructed to wear an ActiGraph 7164 accelerometer (ActiGraph, Shalimar, FL) for 7-consecutive-days attached to their right hip with an elastic band. To obtain hour by hour ST (min/hour) during an average weekday and weekend day, a protocol previously used with the same NHANES dataset to test developmental stage ST disparities by periods of the day was applied [7]. The data were analyzed using the Web App for Processing NHANES Accelerometer Data [8]. S2 Appendix summarizes the features selected to obtain the hour-by-hour ST data from the Web App. After obtaining the hour-by-hour ST, average min/hour were calculated for each period of the day. For weekdays, the periods of the day corresponded to before-school (between 6:00 am and 7:59 am), during school (between 8:00 am and 2:59 pm), afterschool (between 3:00 pm and 5:59 pm), and evening (between 6:00 pm and 9:00 pm). The breakdown of the periods of the day were based on the US average waking time and bedtime since participants were not instructed to report sleep and waking time [9, 10]. Additionally, for the weekday periods, US public school schedules were used to determine during school time as well as afterschool time [11]. For the weekend day, the periods of the day corresponded to morning (between 7:00 am and 11:59 am), afternoon (12:00 pm and 5:59 pm), and evening (6:00 pm and 10:00 pm), and these were based on the weather forecast [12].

Age, annual family income, race/ethnicity and sex were obtained from the demographic data reported via interview. Parents were interviewed if participants were 15-years old or younger; otherwise, participants reported themselves. Age was reported in years. Annual family income was defined as the total family income during the wave of data collection. Participants or parents selected the range that was true for them between $0 and $75,000+ in $5,000 increments. Height and weight were obtained from the NHANES body measurement data and were used to calculate body mass index (kg/*m*^2^).

### Statistical analyses

All analyses were conducted using SAS version 9.4. Green’s formula was used to determine sample size [13]. It was determined that at least 114 participants were needed to test one model with covariates. However, since we proposed a total of 14 regression models at least 1,596 participants (114 x 14 = 1,596) needed to be included to achieve 80% statistical power. To account for multiple testing, we used Bonferroni adjustment for the significance level, i.e., 0.05/14 for testing the regression models.

Descriptive statistics were conducted for age as well as for ST for each period of the day including weekday and weekend. Linear regression analyses were conducted separately for each of the following outcomes: ST min/hour before school, during school, afterschool, and during evenings on weekdays; and ST min/hour during weekend mornings, afternoons, and evenings. For each linear regression, the predictor variables were sex or race/ethnicity. The reference group was Mexican American for race/ethnicity and male for sex. Age, annual family income, and body mass index were adjusting covariates. However, when the predictor variable in the analysis was sex, then race/ethnicity was included as a covariate in the model and vice versa. The regression analyses incorporated the complex sampling design of the NHANES dataset to account for stratification, clustering, and weighting. Details about the linear regression syntax and how we accounted for the complex sampling design are presented in S3 Appendix.

Weekly and monthly differences in ST by sex and race/ethnicity were calculated to identify the periods of the day with the biggest opportunity to reduce sex and race/ethnicity disparities. S1 Fig in the appendix provides details on how to calculate weekly and monthly ST differences. Briefly, daily disparities were projected out into disparities over the course of one week and one month, which we have term “long-term differences.” For all regression analyses, the following conditions were examined: normality, linearity, multicollinearity, autocorrelation, and homoscedasticity. To test normality, the threshold for kurtosis was set up at ± 3 and for skewness at ± 1 [14]. The assumption of linearity was checked by creating partial residual plots to detect outliers and verify that the relationship between the dependent and independent variables is linear [15]. To verify the absence of multicollinearity, a Variance Inflation Factor was used. If the VIF > 100, then multicollinearity exists among the variables included in the linear regression [16]. The Durbin-Watson’s d test was used to determine the presence of autocorrelation and if the d-value was between 1∙5 and 2∙5, we considered that there was no presence of autocorrelation [16]. Lastly, homoscedasticity was verified by creating partial correlation plots since it allows to check if the residuals are equal across the regression line. If this hold true, then this assumption is met [16]. Body mass index was transformed using the natural logarithm function. All other assumptions were met.

Sensitivity analyses were conducted to determine whether the selected timeframes for each period of the day influenced results. Therefore, the same regression analyses were conducted for each period of the day with shift of an hour earlier and a separate analysis with a shift of an hour later. The following periods resulted for the one-hour earlier shift: before school: between 5:00 am and 6:59 am, during school: 7:00 am to 1:59 pm, afterschool: 2:00 pm to 4:59 pm, weekday evening: 5:00 pm to 8:00 pm, weekend morning: 6:00 am to 10:59 am, weekend afternoon: 11:00 am to 4:59 pm, and weekend evening: 5:00 pm to 9:00 pm. The following periods resulted for the one hour later shift: before school: 7:00 am to 8:59 am, during school: 9:00 am to 3:59 pm, afterschool: 4:00 pm to 6:59 pm, weekday evening: 7:00 pm to 10:00 pm, weekend morning: 8:00 am to 12:59 pm, weekend afternoon: 1:00 pm to 6:59 pm, and weekend evening: 7:00 pm to 11:00 pm.

## Results

**Fig 1:**
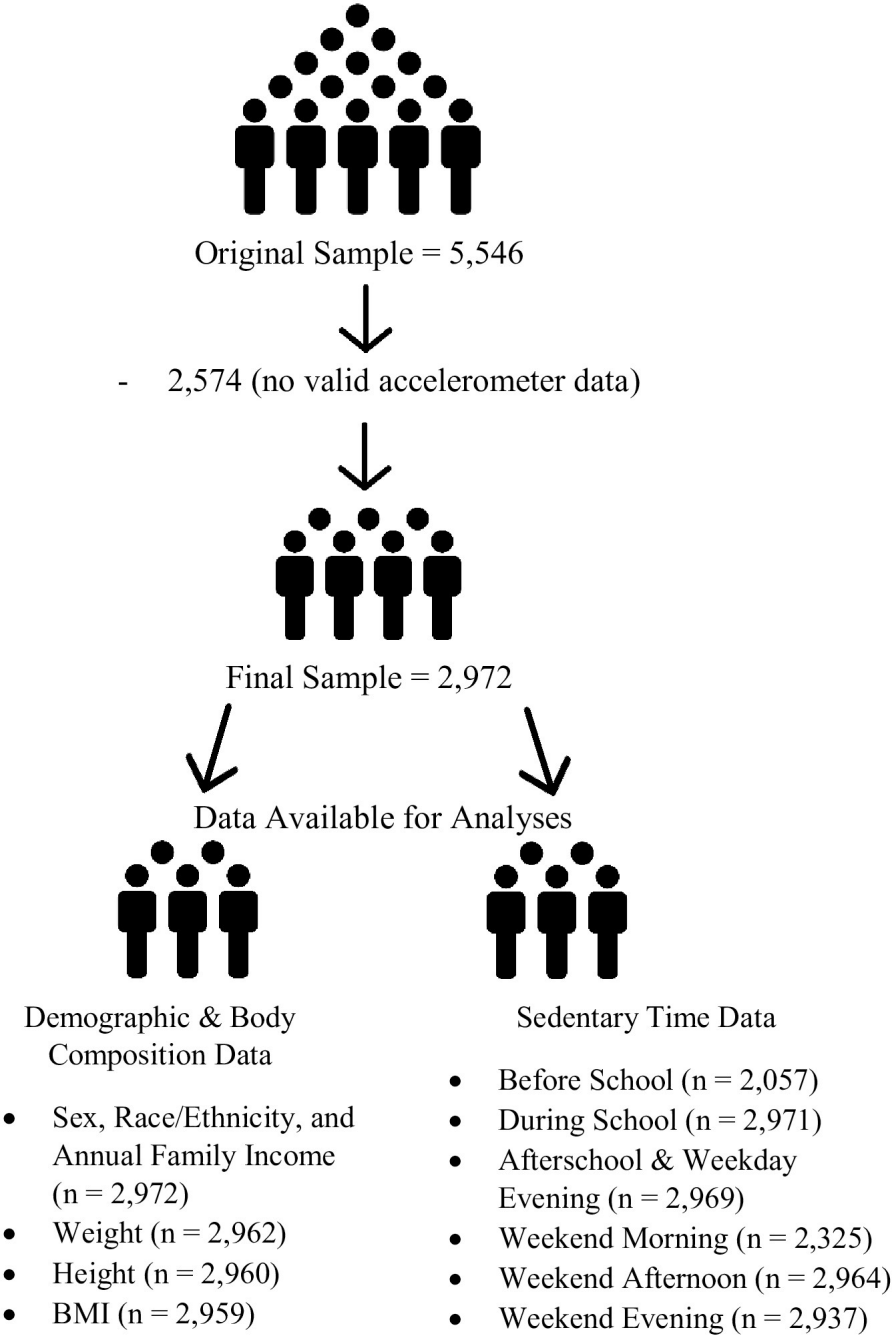
Participants flow diagram.

Fig 1 and S1 Appendix presents details of the final sample. Sensitivity analyses showed no differences across analyses for the periods of the day when shifted one hour earlier and later to test sex disparities in ST. Similarly, no differences across analyses for the periods of the day, besides before school, were reported when shifting one hour earlier and later for each period to test race/ethnic disparities in ST. In the main analysis, race/ethnicity was a significant predictor of before school activity. However, in the two sensitivity analyses (one hour later and one hour earlier) race/ethnicity was not a significant predictor of before school activity, though these were in the same direction in the main analysis. Results, overall, therefore suggest that there may or may not be a difference in before school physical activity by race and ethnicity. Thus, we believe that the proposed timeframe (6:00 AM– 7:59 AM) for before school is not different when shifting the period.

For sex, females were more sedentary than males in the overall week (including weekend days); for race/ethnicity, overall ST by race/ethnicity was significantly different as follows: non-Hispanic White youth spent 448.6 ± 136.0 min/day in ST, Mexican-American youth spent 458.1 ± 129.5 min/day in ST, Other Race–Including Multi-Racial youth spent 461.9 ± 159.1 min/day, Other–Hispanic youth spent 487.8 ± 145.8 min/day, and non-Hispanic Black youth spend 510.6 ± 167.5 min/day in ST.

### Sex disparities in sedentary time

**Fig 2:**
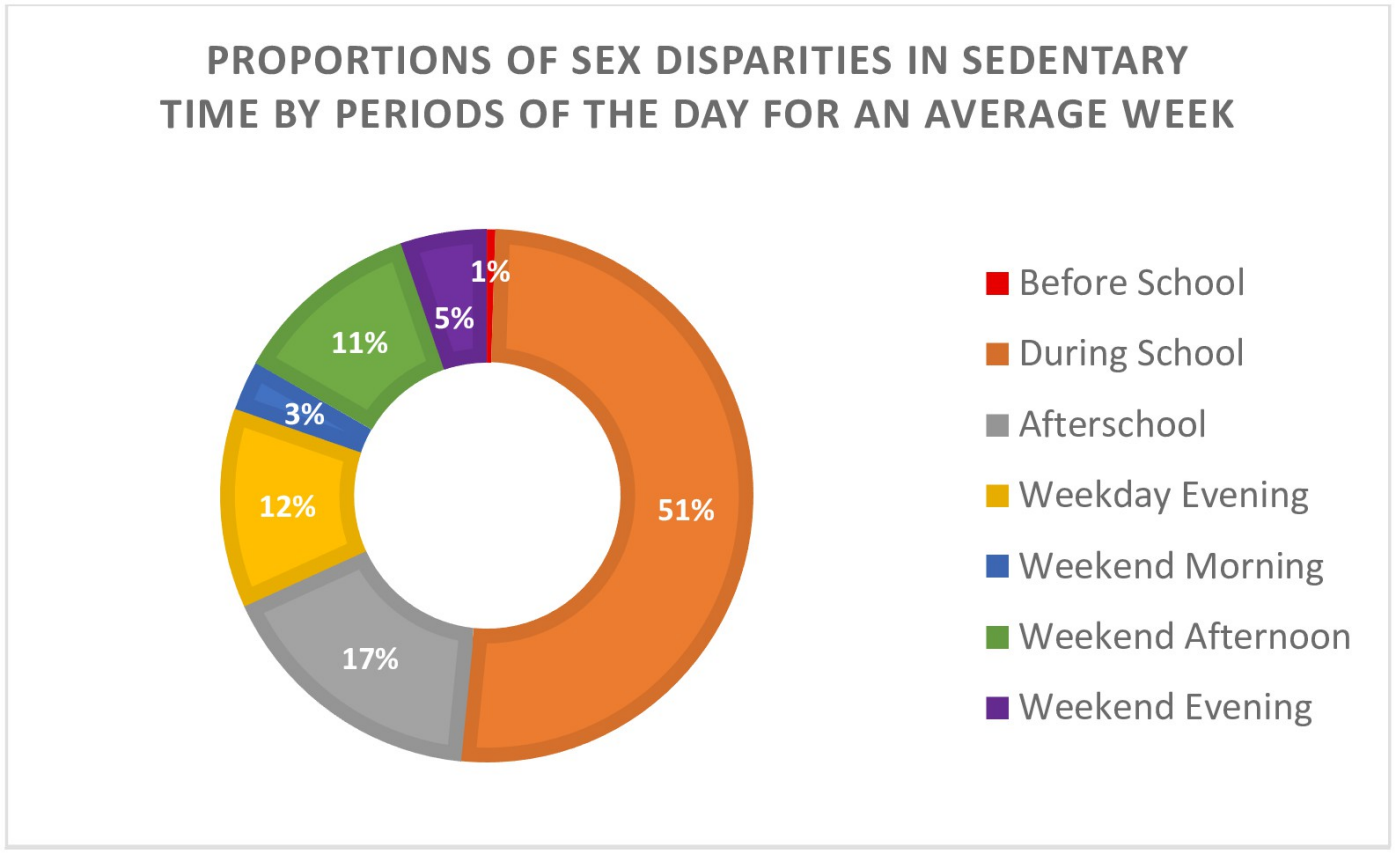
Proportions of sex disparities in sedentary time by periods of the day. Note. To calculate the percentage for each period of the day, the total sum of daily differences from Table 2 was calculated. Secondly, each period of the day was divided by the total sum and then multiply by 100 to obtain the percentage. Example: The total sum of daily differences was 198.7 min/week and the sedentary time sex daily difference for the before school period was 1 min/week. The calculation goes as follows: (1/198.7) x (100) = 0.50 % ≈ 1%.

S4 Appendix presents descriptive statistics of ST during weekday periods and weekend periods by sex as well as the long-term differences in ST by sex. In an average week (including week and weekend days), after calculating the long-term differences in ST by sex, during school and afterschool emerged as the periods with the biggest opportunity to reduce sex disparities in ST. Fig 2 decomposes ST disparities into proportional contributions of each period of the day during an average week. More than half of the sex disparity in ST occurred during school hours despite school hours being almost 1/4 of the hours in a week.

On weekdays, the unadjusted model indicated that sex was not a significant predictor of ST before school (F(2,971, 2,970) = 0.36, p = 0.55). Sex was a significant predictor of ST during school (F(2,957, 2,955) = 66.6, p < 0.0001), afterschool (F(2,971, 2,970) = 25.0, p < 0.0001), and weekday evening period (F(2,671, 2,970) = 12.9, p = 0.001); and all three remained significant predictors of ST after adjustment—during school (F(2,957, 2,949) = 118.5, p < 0.0001), afterschool (F(2,971, 2,963) = 132.2, p < 0.0001), and evening (F(2,971, 2,963) = 106.7, p = 0.004). On weekend days, the unadjusted model indicated that females were more sedentary than males during weekend mornings (F(2,971, 2,970) = 1.5, p = 0.0002) and weekend afternoons (F(2,971, 2,970) = 23.7, p < 0.0001), but not during weekend evenings (F(2,971, 2,970) = 6.5, p = 0.02). After including covariates into the model, sex remained a significant predictor of ST during weekend morning (F(2,971, 2,963) = 35.7, p = 0.002) and weekend afternoon (F(2,971, 2,963) = 92.3, p < 0.0001). Table 1 summarizes unadjusted and adjusted models for the periods of the week and weekend day.

### Race/ethnic disparities in sedentary time

**Fig 3:**
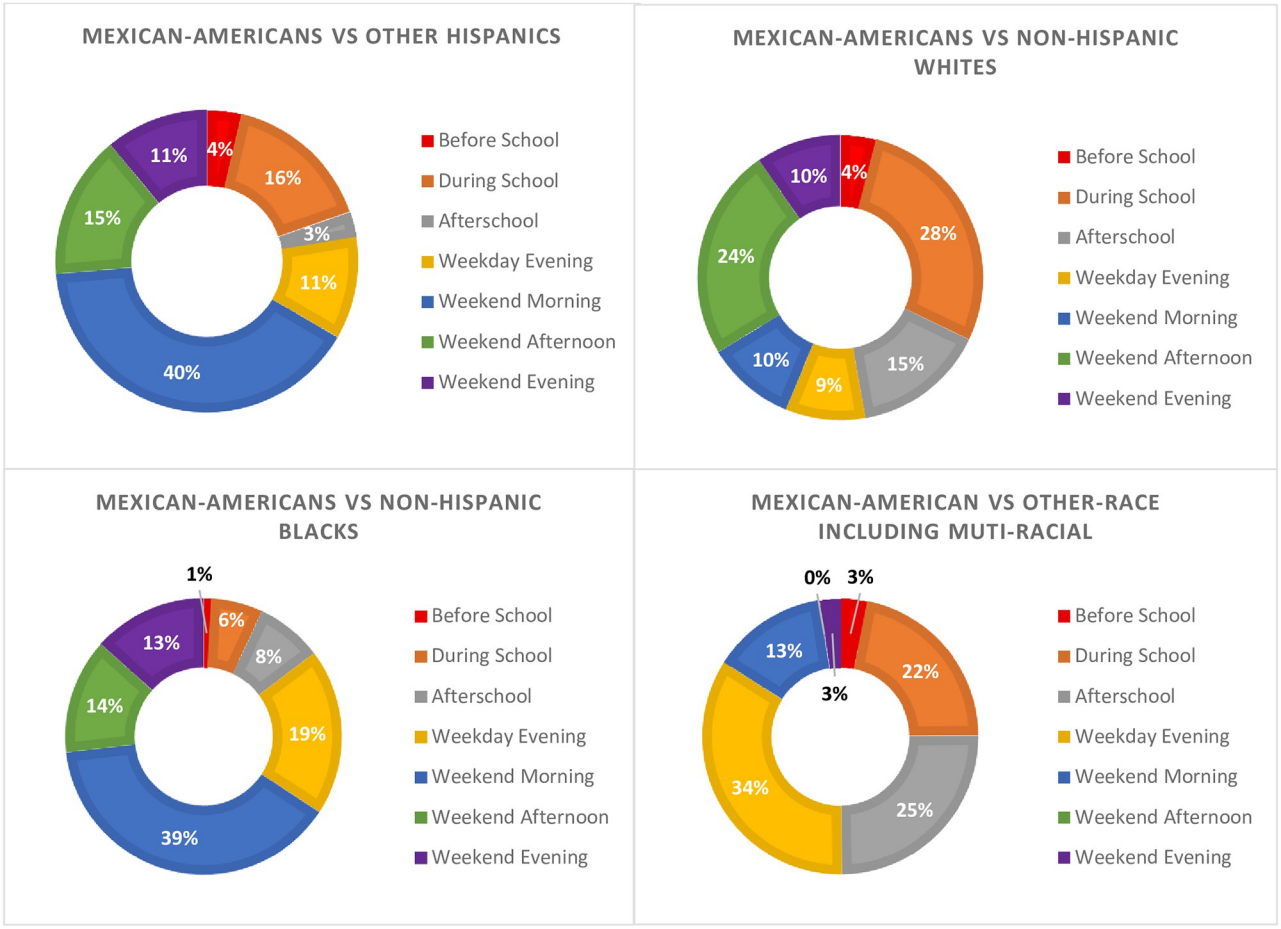
Proportions of race/ethnic disparities in sedentary time by periods of the day.

S5 Appendix presents descriptive statistics of ST during weekday periods (before school, during school, afterschool, and weekday evening) and long-term differences in ST by race/ethnicity. After calculating long term differences in ST by race/ethnicity for an average week (including week and weekend days), based upon the comparison group, different periods of the days emerged as the best opportunity to reduce race/ethnic disparities in ST. Weekend morning was identified as the biggest opportunity to reduce race/ethnic disparities when comparing Mexican Americans to Other Hispanics. When comparing Mexican Americans to Non-Hispanic White, during school emerged as the period with the greatest opportunity to reduce ST disparities. For the comparison between Mexican Americans and Non-Hispanic Blacks, weekend afternoon was identified as the greatest opportunity to reduce ST disparities. Lastly, when comparing Mexican Americans to Other Race–including Multi-Racial, weekend evening emerged as the greatest opportunity to reduce ST disparities. Fig 3 illustrates the proportions of race/ethnic disparities in ST by periods of the day. It shows that 39%–40% of the ST disparities between Mexican-Americans and Non-Hispanic Blacks or Other Hispanics (respectively) occurred during weekend mornings.

On weekdays, the unadjusted models indicated that race/ethnicity was not a significant predictor of ST before school (F(2,971, 2,968) = 0.72, p = 0.6), during school (F(2,971, 2,968) = 0.17 p = 0.1), afterschool (F(2,971, 2,968) = 3.39 p = 0.02), or weekday evening (F(2,971, 2,968) = 4.90, p = 0.01). On weekend days, the unadjusted models indicated that race/ethnicity was a significant predictor of ST during weekend mornings (F(2,971, 2,968) = 11.2, p < 0.0001), but not for weekend afternoon (F(2,971, 2,968) = 2.53, p = 0.06) or weekend evening (F(2,971, 2,968) = 2.83, p = 0.04). Race/ethnicity remained significantly different in ST during weekend mornings after including covariates into the model (F(2,971, 2,963) = 24.6, p < 0.0001;). Since non-Hispanic Blacks were the only race/ethnicity that was significantly different, this implies that identifying as non-Hispanic Black was associated with higher ST during weekend mornings compared to Mexican Americans. Table 2 provides results of regression models for ST at all periods of the weekday and weekend day by race/ethnicity.

**Table 2:** Regression of sedentary time by periods of the a week day and weekend day on race/ethnicity.

Period of the Day	Variables	Unadjusted Model		Adjusted Model		
		Beta (95% CI)	P	Beta (95% CI)	P	*R* ^2^
Before School	non-Hispanic White	-0.3 (-0.9, 0.4)	0.43	-	-	-
	non-Hispanic Black	0.0 (-0.4, 0.5)	0.84	-	-	
	Other Hispanic	0.6 (-0.5, 1.6)	0.26	-	-	
	Other Race–Including Multi Racial	0.0 (-0.7, 0.8)	0.95	-	-	
During School	non-Hispanic White	0.3 (-0.8, 1.3)	0.61	-	-	-
	non-Hispanic Black	0.2 (-0.6, 1.0)	0.62	-	-	
	Other Hispanic	0.7 (-1.2, 2.7)	0.45	-	-	
	Other Race–Including Multi Racial	0.0 (-2.1, 2.2)	1.0	-	-	
Afterschool	non-Hispanic White	1.1 (0.1, 2.1)	0.03	-	-	-
	non-Hispanic Black	-0.6 (-1.6, 0.4)	0.20	-	-	
	Other Hispanic	0.7 (-1.3, 2.7)	0.50	-	-	
	Other Race–Including Multi Racial	-0.2 (-2.9, 2.5)	0.88	-	-	
Weekday Evening	non-Hispanic White	0.2 (-0.9, 1.3)	0.74	-	-	-
	non-Hispanic Black	-1.5 (-3.0, -0.1)	0.04	-	-	
	Other Hispanic	1.6 (-0.5, 3.6)	0.13	-	-	
	Other Race–Including Multi Racial	-0.7 (-3.5, 2.1)	0.61	-	-	
Weekend Morning	non-Hispanic White	0.8 (-1.0, 2.6)	0.35	0.7 (-1.1, 2.4)	0.43	**0.10**
	non-Hispanic Black	**4.3 (2.5, 6.2)**	**<0.0001**	**4.0 (2.2, 5.7)**	**<0.0001**	
	Other Hispanic	4.1 (1.5, 6.8)	0.003	4.5 (2.2, 6.9)	0.0005	
	Other Race–Including Multi Racial	0.3 (-3.8, 4.3)	0.90	2.0 (-2.3, 6.3)	0.36	
Weekend Afternoon	non-Hispanic White	1.5 (0.4, 2.6)	0.01	**-**	**-**	-
	non-Hispanic Black	1.1 (-0.2, 2.4)	0.09	-	-	
	Other Hispanic	2.0 (-0.4, 4.4)	0.11	-	-	
	Other Race–Including Multi Racial	1.2 (-0.9, 3.4)	0.24	-	-	
Weekend Evening	non-Hispanic White	1.2 (-0.1, 2.5)	0.02	-	-	-
	non-Hispanic Black	-1.1 (-2.7, 0.5)	0.18	-	-	
	Other Hispanic	1.3 (-1.6, 4.1)	0.37	-	-	
	Other Race–Including Multi Racial	1.6 (-0.5, 3.6)	0.12	-	-	

**Notes**. In the adjusted model, the following covariates were adjusted: age, income, and BMI. An alpha level of <0.004 was used to determine significance. Only for significant unadjusted models, the unadjusted analyses were conducted and reported.

## Discussion

The purpose of this study was to evaluate sex and racial/ethnic disparities in device-based ST among youth in the US by period of the day using a nationally representative sample of US youth. Findings confirm previous data that support that male youth are less sedentary compared to female youth in different periods of the weekday. Although previous literature has provided similar findings, most of it has been focused on overall ST instead of periods of the day [1]. These ST differences by sex may appear small, but they represent a larger difference when one considers the accumulation of these differences reflected in our long-term differences and the proportions of ST disparities reported for each period of the day. For example, the ST difference by sex within the during school period itself was 2.9 min/hour; however, when extrapolated to min/week (101.5 min/week or ≈ 1.7 hours/week) and min/month (406.0 min/month or ≈ 6.8 hours/month), then it can result in health implications. It has been reported that replacing an hour of ST with moderate to vigorous physical activity results in a 5% reduction of total body fat [17]. The ST disparity by sex was mainly (51%) occurred during school hours alone which is remarkable given that these only compose 21% of total weekly hours. Thus, this setting presents a unique opportunity to reduce ST disparities by sex and better health outcomes for females in the United States.

Although this study did not examine the reason for sex differences in ST during school, it is possible that gender norms shape ST. For instance, a systematic review that examined how gender roles impact physical activity reported that for some girls it is important to participate in activities in which they are allowed to exhibit gender appropriate behaviors and be feminine, but for others it is important to be perceived as strong and feminine at the same time to avoid being perceived as aggressive or lazy by their peers [18]. Thus, the importance of appearance and perceptions for girls may lead them towards sedentary choice more often than boys.

Findings from our study showed that sex was a significant predictor of ST during weekend mornings and afternoons, but not during weekend evenings. It is possible that more males participate in organized sports than females and that most youth organized sport events take place before weekend evenings. It has been reported that 79% of male youth vs 70% of female youth attending third to fifth grade, 77% of male youth vs 72% of female youth attending sixth to eighth grade, 70% of male youth vs 68% of female youth attending ninth to tenth grade, and 69% of male youth vs 64% of female youth attending eleventh to twelfth grade participate in organized sports in the US [19]. Although no youth sport guidelines explicitly state the times in which the youth organized sports should take place, some sports in the US have outlined specific rest guidelines. For instance, the USA Basketball included in their rest guidelines that children between seven to eleven years of age participating in organized basketball should sleep between nine to twelve hours per night and those ≥ 12 years of age should sleep between 8 to 10 hours per night [20]. Thus, to guarantee appropriate sleep time games are not scheduled during late evening and this might partially contribute to our finding.

Race/ethnicity was only a significant predictor of ST during weekend mornings after controlling for covariates. Particularly, it was reported that Mexican Americans were less sedentary than non-Hispanic Blacks during weekend mornings. Even though we do not fully understand the underlying mechanisms of this disparity in ST, it might be the case that cultural differences can partially explain it. For many Latino families providing an environment that promotes loyalty and reciprocity among family members–known as *familismo*–tends to be a core value which can influence the family routines and its members’ behavior [21]. For example, Latino family members (including nuclear and extended family members) tend to do daily activities together such as preparing, eating, and cleaning up after breakfast together and even attending extracurricular activity related to a child such as sport event or recital [21]. This may influence the amount of ST during weekend morning compared to other racial/ethnic groups.

After calculating long term differences and examining the proportion of ST disparities by period of the day, the period that emerged as the largest opportunity to reduce ST disparities by race/ethnicity was the weekend mornings. Even though these differences seem small, when looking at the long-term differences these may have large health impacts. For instance, the weekend morning difference in ST between Mexican Americans and Non-Hispanic Blacks was 4.5 min/period, but when extrapolated to weekly differences this is a difference of 45.0 min/week. The weekend morning difference represents 39% of the ST disparities in an average week (week and weekend days) between Mexican Americans and non-Hispanic Blacks. Similarly, the weekend morning difference in ST between Mexican Americans and other Hispanics was of 4.4 min/period but when extrapolated to weekly differences this is a difference of 44.0 min/week. In this case, the weekend morning difference represents a 40% of the ST disparities in an average week (week and weekend days). In both cases, the weekly difference is near to an hour, and it is known that substituting one hour of ST for one hour of light physical activity can result in better cardiometabolic health outcomes such as lower HDL cholesterol and diastolic blood pressure [22].

The current study provides insight for interventionists about which periods of the day should be targeted to reduce ST disparities by sex and race/ethnicity. However, it is not exempt of limitations since the cross-sectional design by nature does not allow us to determine causality. Regression analyses revealed that sex explained 27%, 29%, 23%, 15%, and 1% of the variance in ST for during school, afterschool, weekday evening, weekend morning, and weekend afternoon, respectively. Secondly, r-squared values showed race/ethnicity explained 10% of the variance in ST during weekend mornings. All r-squared values are weak to moderate, which can be explained by not considering other factors such as sleep time and ST type (e.g., screen time, educational ST). Both variables have shown to affect ST levels and differences by race/ethnicity [23]. Authors were not able to obtain sleep time or ST type data since these variables are not part of the 2003–2004 and 2005–2006 NHANES dataset. In addition, we were not able to account for confounders during school time (i.e., schedule and physical education class) because the NHANES data set did not collect this information.

Fourth, the study only considered sex and not gender, which means that it did not consider the societal expectations of being male or female, though these overlap substantially. Fifth, the data was collected between 2003 and 2006, which raises questions about the relevance of the findings in 2023. Although recent studies have shown an increase in ST [24, 25], it is a small increase. For instance, a study that assessed ST with accelerometers among children and adolescents living in Norway during years 2005, 2011, and 2018 reported a 6% increase in ST among boys and 1% increase in ST among girls when comparing 2005 vs 2018 [24]. Moreover, a study in 2017 that recorded device-based ST among a sample of 8–12 years old US children reported that the sample spent 8.3 ± 2.1 hours/day being sedentary [25]. If this ST data (8.3 hours/day) is compared with the 2003–2006 NHANES data (7.9 hours/day), then there has been an increase in ST of approximately 2% in the US.

Lastly, results should be interpreted with caution since this is a secondary analysis of a publicly available dataset, and some factors were out of our control. For instance, the device used to determine ST cannot distinguish between sitting and standing still and we cannot determine whether accelerometers were worn during school year or summer. Hence, it is possible that some participants were standing and this was considered as ST and that data was collected in different points (school days vs summer days). In both cases, the rate of this occurring is likely similar between groups, thus; the factor is equal between groups being compared and has been washed out. To our knowledge this is the first study to provide insights about ST data by periods of the day by sex and race/ethnicity using a US representative youth sample.

Understanding of the periods of the day during which disparities occur could provide meaningful information for future interventions meant to reduce sex and race/ethnic disparities in ST. This is because periods of the day also correspond with variation in setting and supervising adult. Our data suggest that weekend mornings, when children are home with parents, are a promising target for reducing ST disparities by race/ethnicity. In contrast, school and after-school hours, when children are under the supervision of educators and after-school program staff, represent a promising target for reducing sex disparities in ST. The opposite is also true, it may not make sense to intervene on a sociodemographic disparity during periods of the day that no disparity exists.

## Supporting information

S1 Appendix Participants distribution, descriptive characteristics, & overall sedentary time by sex & by race/ethnicity. (PDF)

S2 Appendix Accelerometer data processing specifications. (DOCX)

S3 Appendix Description of the linear regression syntax for SAS. (DOCX)

S4 Appendix Patterns of sedentary time during an average weekday & weekend day by sex. (DOCX)

S5 Appendix Patterns of sedentary time during an average weekday & weekend day by race/ethnicity. (DOCX)

S1 Fig How to calculate long term differences in sedentary time. (DOCX)
