# Supplementary material for: Sociodemographic disparities in sedentary time among US youth vary by period of the day
Source: PLoS One. 2024 Jan 5;19(1):e0296515. doi: 10.1371/journal.pone.0296515 (PMC10769050; doi:10.1371/journal.pone.0296515)
Supplement: S1 Appendix — (PDF) [file pone.0296515.s001.pdf]

Table 1. Participants Distribution, Descriptive Characteristics, & Overall Sedentary Time by Sex & by Race/Ethnicity

**Participants Distribution by Race/Ethnicity and Sex**						
Race/Ethnicity	Total N (%)		Female N (%)		Male N (%)	
Mexican-American	1,033 (34.8%)		522 (17.6%)		511 (17.2%)	
Non-Hispanic Black	1,008 (33.9%)		477 (16.0%)		531 (17.9%)	
Non-Hispanic White	712 (24.0%)		373 (12.6%)		339 (11.4%)	
Other Hispanic	84 (2.8%)		41 (1.4%)		43 (1.4%)	
Other Race – Including Multi Racial	135 (4.5%)		72 (2.4%)		63 (2.1%)	
		**Sex**				
		Female (N = 1,485)	Male (N = 1,487)	Difference		
Min/Day Overall ST (mean ± SD)		481.2 ± 145.4	468.0 ± 152.3	**13.2***		
Age (years)		12.4 ± 3.6	12.5 ± 3.6	0.03		
BMI (kg/m^2)		21.8 ± 5.9	21.2 ± 5.5	**0.6***		
Annual Family Income	Under $20,000 (n)	396	369	N/A		
	$20,000 and over (n)	1,055	1,078			
		**Race/Ethnicity**				
	Mexican American (N = 1,033)	non-Hispanic Black (N = 1,008)	non-Hispanic White (N = 712)	Other Hispanic (N = 84)	Other Race (N = 135)	P-value
Min/Day Overall ST (Mean ±SD)	458.1 ± 129.5	510.6 ± 167.5	448.6 ± 136.0	487.8 ± 145.8	461.9 ± 159.1	**<0.0001**
Age (years)	12.2 ± 3.5	12.9 ± 3.5	12.4 ± 3.7	12.6 ± 3.7	11.3 ± 3.7	**<0.0001**
BMI (kg/m^2)	21.6 ± 5.5	22.2 ± 6.3	20.9 ± 5.2	21.7 ± 4.9	19.0 ± 4.5	**<0.0001**
Annual Family Income: Under $20,000 (n)	301	336	92	20	16	N/A
Annual Family Income: $20,000 and over (n)	696	644	611	63	119	

Notes. ST = sedentary time, SD = standard deviation, BMI = body mass index, N/A = Not Applicable, *p<0.05
