# Supplementary material for: Sociodemographic disparities in sedentary time among US youth vary by period of the day
Source: PLoS One. 2024 Jan 5;19(1):e0296515. doi: 10.1371/journal.pone.0296515 (PMC10769050; doi:10.1371/journal.pone.0296515)
Supplement: S3 Appendix — (DOCX) [file pone.0296515.s003.docx]

| **Appendix S3. Description of the Linear Regression Syntax for SAS** | |
| --- | --- |
| Syntax for Sex & Race/Ethnicity as Predictors Separately | Description |
| **PROC SURVEYREG data= WORK.New2 nomcar;**  **STRATA sdmvstra;**  **CLUSTER sdmvpsu;**  **WEIGHT wtmec4yr;**  **MODEL STDuringSchool=Female/CLPARM vadjust=none;**  **TITLE ‘Unadjusted Linear Regression Model for Weekday Sedentary Time Before School and Sex: NHANES 2003-2006’;**  **run;**  **PROC SURVEYREG data= WORK.New2 nomcar;**  **STRATA sdmvstra;**  **CLUSTER sdmvpsu;**  **WEIGHT wtmec4yr;**  **MODEL STDuringSchool=** **Non_Hispanic_White Non_Hispanic_Black Other_Hispanic Other_Race_Multi_Racial/CLPARM vadjust=none;**  **TITLE ‘Unadjusted Linear Regression Model for Weekday Sedentary Time Before School and Race: NHANES 2003-2006’;**  **run;** | **PROC SURVEYREG**: This statement specifies the input data set and the method to obtain the variance estimation. |
|  | **data**: Identifies the data set that will be used in the analysis |
|  | **nomcar**: This statement is used to compute variance estimate by treating missing values as a subpopulation, which means that the entire population includes missing and non-missing values. |
|  | **STRATA**: This statement indicates that within the data set there is a certain variable that determine levels |
|  | **sdmvstra**: Name for the continuous NHANES variable that allows researchers to account for the stratification effects. In this case, most stratum contain two primary sampling units which are defined by geographic and proportions of minority populations. |
|  | **CLUSTER**: This statement was used to identify the clusters in a sample clustered design. |
|  | **sdmvpsu**: Name of the variable that represents the sample cluster within NHANES. This dataset used a sampling procedure of four stages. The first stage was the primary sampling unit which consisted of groups of contiguous counties. The second stage was dividing those counties into segments or city blocks. The third stage refers to selecting households within the segments. Finally, the last stage, selected individuals within the household. Therefore, the need to account for clusters. |
|  | **WEIGHT**: This statement allows for assignment of a sample weight to each participant. It accounts for unequal probability of nonresponse interviews, sampling, and adjustment to population control totals. |
|  | **wtmec4yr**: This variable allows researchers to assign a sample weight to each person; resulting in nationally representative data. It is a measure of the number of people in the population represented by that sample person in the dataset that adjusts for unequal probability of selection, nonresponse, and independent population controls. |
|  | **MODEL**: The statement allows you to define your equation or statistical model based on the response variable. |
|  | **CLPARM**: This function allows to obtain the 95% confidence interval. |
|  | **vadjust**=none: In this case, no adjustment using the degrees of freedom were used in the variance estimation. |
|  | **TITLE**: This statement allows researchers to assign a title to their output. |
|  | |
