# Supplementary material for: Sociodemographic disparities in sedentary time among US youth vary by period of the day
Source: PLoS One. 2024 Jan 5;19(1):e0296515. doi: 10.1371/journal.pone.0296515 (PMC10769050; doi:10.1371/journal.pone.0296515)
Supplement: S4 Appendix — (DOCX) [file pone.0296515.s004.docx]

| **Appendix S4. Patterns of Sedentary Time during an Average Weekday & Weekend Day by Sex** | | | | | |
| --- | --- | --- | --- | --- | --- |
| Period of the Day | | Sample Size  F M | Female  (mean ± SD) | Male  (mean ± SD) | Difference (min/hour) |
| Before School (min/hour) | | 1,025 1,032 | 36.0 ± 12.5 | 35.9 ± 13.6 | 0.1 |
| During School (min/hour) | | 1,485 1,486 | 33.9 ± 7.9 | 31.0 ± 8.5 | **2.9*** |
| Afterschool (min/hour) | | 1,483 1,486 | 28.2 ± 8.4 | 26.0 ± 8.5 | **2.2*** |
| Weekday Evening (min/hour) | | 1,484 1,485 | 29.6 ± 8.7 | 28.0 ± 9.0 | **1.6*** |
| Weekend Morning (min/hour) | | 1,148 1,177 | 34.2 ± 13.3 | 33.6 ± 13.9 | **0.6*** |
| Weekend Afternoon (min/hour) | | 1,483 1,481 | 28.4 ± 9.9 | 26.5 ± 9.9 | **1.9*** |
| Weekend Evening (min/hour) | | 1,466 1,471 | 30.0 ± 10.6 | 28.7 ± 10.9 | 1.3 |
|  | **Long Term Differences in ST by Periods of the Day** | | | | |
| Period of the Day | | | Daily Difference (min/day) | Weekly Difference (min/week) | Monthly Difference  (min/month) |
| 3Before School  During School  Afterschool  Weekday Evening  Weekend Morning  Weekend Afternoon  Weekend Evening | | | 0.2 | 1.0 | 4.0 |
|  |  |  | 20.3 | 101.5 | 406.0 |
|  |  |  | 6.6 | 33.0 | 132.0 |
|  |  |  | 4.8 | 24.0 | 96.0 |
|  |  |  | 3.0 | 6.0 | 24.0 |
|  |  |  | 11.4 | 22.8 | 91.2 |
|  |  |  | 5.2 | 10.4 | 41.6 |
| Notes. F = Female, M = Male, * Significant difference, SD = Standard Deviation, Before School = between 6:00 am and 7:59 am), During School = between 8:00 am and 2:59 pm, Afterschool = between 3:00 pm and 5:59 pm, Weekday Evening = between 6:00 pm and 9:00 pm, Weekend Morning = between 7:00 am and 11:59 am, Weekend Afternoon = 12:00 pm and 5:59 pm, and Weekend Evening = 6:00 pm and 10:00 pm. | | | | | |
