# Supplementary material for: Sociodemographic disparities in sedentary time among US youth vary by period of the day
Source: PLoS One. 2024 Jan 5;19(1):e0296515. doi: 10.1371/journal.pone.0296515 (PMC10769050; doi:10.1371/journal.pone.0296515)
Supplement: S5 Appendix — (DOCX) [file pone.0296515.s005.docx]

| **Appendix S5. Patterns of Sedentary Time during an Average Weekday & Weekend Day by Race/Ethnicity** | | | | | |
| --- | --- | --- | --- | --- | --- |
| Period of the Day | Mexican-American  (N: mean ± SD) | Other Hispanic  (N: mean ± SD) | Non-Hispanic White  (N: mean ± SD) | Non-Hispanic Black  (N: mean ± SD) | Other Race – Including Multi-Racial  (N: mean ± SD) |
| Before School (min/hour) | 762: 35.1 ± 3.7 | 62: 35.5 ± 3.4 | 469: 34.9 ± 3.7 | 812: 35.2 ± 3.5 | 87: 34.8 ± 4.2 |
| During School (min/hour) | 1,033: 32.5 ± 8.1 | 84: 32.0 ± 7.8 | 711: 32.1 ± 8.5 | 1,008: 32.7 ± 8.3 | 135: 31.9 ± 8.9 |
| Afterschool (min/hour) | 1,033: 27.3 ± 8.0 | 84: 27.1 ± 7.2 | 711: 27.8 ± 9.0 | 1,007: 26.7 ± 8.5 | 134: 25.7 ± 9.8 |
| Weekday Evening (min/hour) | 1,032: 29.5 ± 8.6 | 84: 30.3 ± 8.7 | 712: 29.2 ± 9.2 | 1,007: 28.0 ± 8.8 | 134: 27.3 ± 9.8 |
| Weekend Morning (min/hour) | **809: 32.1 ± 13.0*** | 68: 36.5 ± 11.0 | 570: 32.6 ± 13.1 | **768: 36.6 ± 14.2*** | 110: 33.4 ± 14.5 |
| Weekend Afternoon (min/hour) | 1,032: 26.7 ± 9.3 | 84: 28.1 ± 9.3 | 711: 27.7 ± 10.3 | 1,004: 28.0 ± 10.1 | 133: 26.7 ± 11.0 |
| Weekend Evening (min/hour) | 1,021: 29.5 ± 10.3 | 83: 31.0 ± 11.2 | 707: 30.1 ± 10.8 | 995: 28.4 ± 11.1 | 131: 29.2 ± 10.9 |
| **Long Term Differences in ST by Periods of the Day** | | | | | |
| Comparison Groups | Period of the Day | Difference within the Period (min/period) | Daily Difference (min/day) | Weekly Difference (min/week) | Monthly Difference  (min/month) |
| Mexican- American vs Other Hispanics | Before School | 0.4 | 0.8 | 4.0 | 16.0 |
|  | During School | 0.5 | 3.5 | 17.5 | 70.0 |
|  | Afterschool | 0.2 | 0.6 | 3.0 | 12.0 |
|  | Weekday Evening | 0.8 | 2.4 | 12.0 | 48.0 |
|  | Weekend Morning | 4.4 | 22.0 | 44.0 | 176.0 |
|  | Weekend Afternoon | 1.4 | 8.4 | 16.8 | 67.2 |
|  | Weekend Evening | 1.5 | 6.0 | 12.0 | 48.0 |
| Mexican-American vs Non-Hispanic White | Before School | 0.2 | 0.4 | 2 | 8.0 |
|  | During School | 0.4 | 2.8 | 14 | 56.0 |
|  | Afterschool | 0.5 | 1.5 | 7.5 | 30.0 |
|  | Weekday Evening | 0.3 | 0.9 | 4.5 | 18.0 |
|  | Weekend Morning | 0.5 | 2.5 | 5 | 20.0 |
|  | Weekend Afternoon | 1 | 6 | 12 | 48.0 |
|  | Weekend Evening | 0.6 | 2.4 | 4.8 | 19.2 |
| Mexican-American vs Non-Hispanic Black | Before School | 0.1 | 0.2 | 1 | 4.0 |
|  | During School | 0.2 | 1.4 | 7 | 28.0 |
|  | Afterschool | 0.6 | 1.8 | 9 | 36.0 |
|  | Weekday Evening | 1.5 | 4.5 | 22.5 | 90.0 |
|  | Weekend Morning | 4.5 | 22.5 | 45 | 180.0 |
|  | Weekend Afternoon | 1.3 | 7.8 | 15.6 | 62.4 |
|  | Weekend Evening | 1.1 | 7.7 | 15.4 | 61.6 |
| Mexican American vs Other Race – Including Multi-Racial | Before School | 0.3 | 0.6 | 3 | 12.0 |
|  | During School | 0.6 | 4.2 | 21 | 84.0 |
|  | Afterschool | 1.6 | 4.8 | 24 | 96.0 |
|  | Weekday Evening | 2.2 | 6.6 | 33 | 132.0 |
|  | Weekend Morning | 1.3 | 6.5 | 13 | 52.0 |
|  | Weekend Afternoon | 0 | 0 | 0 | 0 |
|  | Weekend Evening | 0.3 | 1.2 | 2.4 | 9.6 |
| Notes. * Significant difference between race/ethnic groups, SD = Standard Deviation, Before School = between 6:00 am and 7:59 am), During School = between 8:00 am and 2:59 pm, Afterschool = between 3:00 pm and 5:59 pm, Weekday Evening = between 6:00 pm and 9:00 pm, Weekend Morning = between 7:00 am and 11:59 am, Weekend Afternoon = 12:00 pm and 5:59 pm, and Weekend Evening = 6:00 pm and 10:00 pm. | | | | | |
