# Supplementary material for: Sociodemographic disparities in sedentary time among US youth vary by period of the day
Source: PLoS One. 2024 Jan 5;19(1):e0296515. doi: 10.1371/journal.pone.0296515 (PMC10769050; doi:10.1371/journal.pone.0296515)
Supplement: S1 Fig — (DOCX) [file pone.0296515.s006.docx]

Figure S1. How to Calculate Long Term Differences in Sedentary Time

Examples

Steps


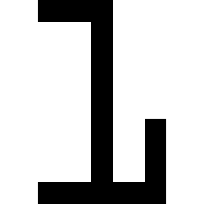


We know that the weekly ST difference by sex for before school is 1.0 min/week.

Then, the calculation goes as follows:

**(1 min/week) x (4) = 4.0 min/month**

Calculate the monthly difference.

For this, you will multiply the weekly difference times four (assuming that a month has 4 weeks).


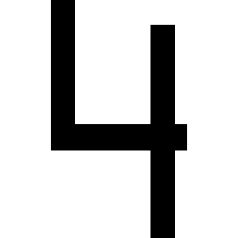


Calculate the weekly difference.

For this, you will multiply the daily difference times five (representing the 5 weekdays).

We know that the daily ST difference by sex for before school is 0.2 min/day.

Then, the calculation goes as follows:

**(0.2 min/day) x (5) = 1 min/week**


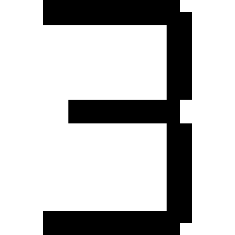


Calculate the daily difference.

For this, you will multiply the period difference by the amount of hours that a particular period last.

We know that the ST difference by sex for before school is 0.1 min/hour and that the before school period starts at 5:00 AM and it is over at 6:59 AM, which means it last 2 hours. Then, the calculation goes as follows:

**(0.1 min/hour) x (2) = 0.2 min/day**


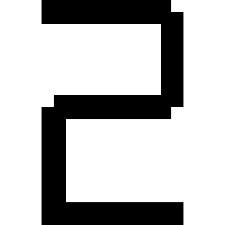


If the mean ST before school for males (reference group) was 35.9 min/hour and the mean ST before school for females was 36.0 min/hour. Then, the calculation goes as follows:

**|35.9 – 36.0| = 0.1 min/hour**

Calculate the period of the day difference. To do so, calculate the absolute value of the difference between the mean ST min/hour of the reference group and the group you which to compare to.
